# Preoperative risk assessment improves biomarker detection for predicting acute kidney injury after cardiac surgery

**DOI:** 10.1371/journal.pone.0203447

**Published:** 2018-09-04

**Authors:** Cheng-Chia Lee, Chih-Hsiang Chang, Shao-Wei Chen, Pei-Chun Fan, Su-Wei Chang, Yi-Ting Chen, Yu-Yun Nan, Pyng-Jing Lin, Feng-Chun Tsai

**Affiliations:** 1 Kidney Research Center, Department of Nephrology, Chang Gung Memorial Hospital, Linkou branch, College of Medicine, Chang Gung University, Taoyuan, Taiwan; 2 Graduate Institute of Clinical Medical Sciences, College of Medicine, Chang Gung University, Taoyuan, Taiwan; 3 Department of Cardiothoracic and Vascular Surgery, Chang Gung Memorial Hospital, Linkou Branch, College of Medicine, Chang Gung University, Taoyuan, Taiwan; 4 Clinical Informatics and Medical Statistics Research Center, College of Medicine, Chang Gung University, Taoyuan, Taiwan; 5 Division of Allergy, Asthma, and Rheumatology, Department of Pediatrics, Chang Gung Memorial Hospital, Taoyuan, Taiwan; 6 Department of Biomedical Sciences, Chang Gung University, Taoyuan, Taiwan; University of Sao Paulo Medical School, BRAZIL

## Abstract

**Background:**

Although urinary neutrophil gelatinase-associated lipocalin (NGAL) has emerged as a promising biomarker for the early detection of kidney injury, previous studies of adult patients who underwent cardiac surgery have reported only moderate discrimination. The age, creatinine, and ejection fraction (ACEF) score is a preoperative validated risk model with satisfactory accuracy for predicting AKI following cardiac surgery. It remains unknown whether combining preoperative risk assessment through ACEF scores followed by urinary NGAL test in a population of high-risk individuals is an optimal approach with improved predictive performance.

**Material and methods:**

A total of 177 consecutive patients who underwent cardiac surgery were enrolled. Clinical characteristics, prognostic model scores, and outcomes were assessed. Urinary NGAL were examined within 6 hours after cardiac surgery. Patients were stratified according to preoperative ACEF scores, and comparisons were made using the area under the receiver operator characteristic curve (AUROC) for the prediction of AKI.

**Results:**

A total of 45.8% (81/177) of the patients had AKI. As expected, patients with ACEF scores ≥ 1.1 were older and more likely to have class III or IV heart failure. They were also more likely to have diabetes mellitus, myocardial infarction, and peripheral arterial disease. Urinary NGAL alone moderately predicted AKI, with an AUROC of 0.732. Risk stratification by ACEF scores ≥ 1.1 substantially improved the AUROC of urinary NGAL to 0.873 (95% confidence interval, 0.784–0.961; *P* < .001).

**Conclusions:**

Risk stratification by preoperative ACEF scores ≥ 1.1, followed by postoperative urinary NGAL, provides more satisfactory risk discrimination than does urinary NGAL alone for the early detection of AKI after cardiac surgery. Future studies should investigate whether this strategy could improve the outcomes and cost-effectiveness of care in patients undergoing cardiac surgery.

## Introduction

Acute kidney injury (AKI) occurs in 10%–50% of patients undergoing cardiac surgery.[1–5] This statistical variation is largely due to heterogeneous study populations and the use of different criteria to define AKI. Notably, a recent meta-analysis reported that irrespective of the different guideline-based AKI definitions, the presence of AKI in patients undergoing cardiac surgery is consistently associated with a 2- to 4-fold increase in the risk of early mortality.[6] Furthermore, a clear dose–response relationship exists between the severity of AKI and poor outcomes.[2, 6, 7]

Despite decades of research, the clinical management of AKI mainly focused on hemodynamic manipulations, attenuating ischemia and supportive care.[8] A major challenge to developing effective interventions for AKI has been the limited ability to detect this condition in its early stages. Hence, several proteins have been identified as sensitive and specific biomarkers for detecting AKI.[9–11] A frequently studied biomarker is urinary neutrophil gelatinase-associated lipocalin (NGAL), which has demonstrated promise in detecting postoperative AKI earlier than serum creatinine can.[11–13] However, compared with the excellent discrimination reported in pediatric patients, a recent meta-analysis of studies of urinary NGAL in adults reported only moderate discrimination, with an area under the receiver operating characteristic curve (AUROC) of 0.72 (95% confidence interval, 0.66–0.79).[14] Its comparatively high costs render it less useful at present than serum creatinine as a routine clinical tool. Therefore, we examined whether a diagnostic flow chart with stratification by clinical risk assessment as the first line, followed by the urinary NGAL test in a population of high-risk individuals, can improve overall performance.

Numerous clinically validated risk models for cardiac surgery are currently in use. The age, creatinine, and ejection fraction (ACEF) score was initially developed to simplify bedside evaluation by using only 3 critical variables for predicting mortality in patients undergoing coronary artery bypass grafting (CABG).[15] According to the “law of parsimony” for excluding excessive variables that might result in overfitting and the risk of multicollinearity, the ACEF score exhibits similar or superior accuracy to that of a previously established complex model for stratifying mortality risk.[15–17] Our recent studies have demonstrated that ACEF scores can also accurately identify the risk of postoperative AKI in patients undergoing either valve surgery [4] or CABG.[5] We concluded that this extremely user-friendly ACEF score might be an optimal risk assessment tool. The aim of this investigation was to evaluate the discrimination ability of urinary NGAL after risk stratification by ACEF scores.

## Materials and methods

### Study participants and design

This observational investigation was performed in the cardiac surgery intensive care unit (ICU) of a tertiary care referral center in Taiwan between July 2014 and February 2015. The study protocol was approved by the institutional review board of the Chang Gung Memorial Hospital (No. 103-1993B). Consecutive patients who provided informed consent and who were admitted to the ICU immediately after cardiac surgery were enrolled. Patients who had estimated glomerular filtration rate (eGFR) < 30 mL/min/1.73m^2^, were receiving dialysis, were aged <20 years, had confirmed AKI before surgery, or reported any prior organ transplantation were excluded.

### Data collection and definition

Demographic data, clinical characteristics, echocardiographic data, and routine biochemistry test results were collected in a prospective database and retrospectively evaluated. Comorbidities were evaluated according to the following definitions: myocardial infarction was defined according to the 2007 Expert Consensus Document of Circulation, European Heart Journal; Peripheral arterial disease (PAD) was defined as a confirmed ankle–brachial index < 0.9, diagnosis established through duplex ultrasonography, and need for medication; and chronic obstructive pulmonary disease was confirmed through a spirometer test or a medical history and need for treatment.

Baseline serum creatinine was determined as the concentration measured within one week before surgery. If multiple values were available during this week, the creatinine level was defined as the lowest value. If unavailable, the value measured at the steady state within 2 months before admission was used as a surrogate baseline. After cardiac surgery, we measured serum creatinine levels every morning for up to 1 week. The ACEF scores were calculated as age (years)/ejection fraction (EF)% + 1 (if creatinine > 2.0 mg/dL).[15] The EF was measured through 2-dimensional echocardiography before surgery.

### Outcome definition

To determine the predictive value of biomarkers for AKI, the primary outcome was the development of AKI within 7 days after cardiac surgery. Based on the Kidney Disease Improving Global Outcomes (KDIGO) Clinical Practice Guidelines for Acute Kidney Injury, AKI was confirmed under any of the following conditions: serum creatinine levels ≥ 0.3 mg/dL within 48 hours or a ≥1.5-fold increase in serum creatinine levels from baseline within 7 days.[18] The cutoff value of preoperative ACEF scores for predicting AKI in our earlier studies was 1.2 for CABG and 1.1 for valve surgery, according to the optimal Youden index and overall accuracy.[4, 5] To increase the sensitivity of this ACEF score for screening high-risk group for AKI, we applied a cutoff value of 1.1. Other outcome measures included in-hospital mortality, renal replacement therapy, duration of ventilation, and length of ICU stay.

### Measurement of NGAL levels

Fresh urine samples were collected within 6 hours postoperatively in nonheparinized tubes via an indwelling Foley catheter and were then centrifuged at 5000 × g for 30 minutes at 4°C to remove cells and debris. The clarified supernatants were stored at −80°C before measurement. Urinary NGAL levels were measured in duplicate by using commercially available enzyme-linked immunosorbent assay kits, according to the manufacturer’s instructions (R&D Systems, DLCN20; Mpls, USA). The intra-assay coefficient of variability for urine NGAL was 8.37%.

### Statistical analysis

We compared the continuous variables between groups (i.e., AKI vs. non-AKI or ACEF ≥ 1.1 vs. ACEF < 1.1) using the student t-test. Categorical data between groups were compared using the Fisher exact test. The performance of NGAL in discriminating AKI was assessed using the AUROC. We then performed the ROC analysis stratified by ACEF levels and applied a nonparametric approach[19] to compare the AUROCs of NGAL at different ACEF levels. To determine the optimal cut-off value for NGAL in discriminating AKI, we estimated Youden index, which is defined as (sensitivity + specificity) - 1. The optimal cut-off value for NGAL is that its corresponding Youden index achieves the maximum because it is the point that the highest sensitivity and specificity values occur at the same time. Finally, after incorporating the 40% AKI incidence for individuals who underwent cardiac surgery in Taiwan,[4,5,20] positive and negative predictive values were calculated, given the optimal cutoff value of NGAL. A two-sided P value < 0.05 was considered to be statistically significant. No adjustment of multiple testing (multiplicity) was made in this study. The statistical analyses were conducted using SPSS 22 (IBM SPSS, Armonk, NY: IBM Corp).

## Results

### Study population characteristics: Non-AKI versus AKI groups

A total of 177 consecutive patients who underwent cardiac surgery between July 2014 and February 2015 were enrolled in the study. Postoperative AKI occurred in 81 (45.8%) patients. Among the study patients, 73 (41.2%) had preoperative ACEF scores ≥ 1.1. Clinical characteristics and laboratory data of all the study patients and those with ACEF scores ≥ 1.1, stratified by the presence of AKI, are summarized in Table 1. There was no significant difference in age, sex, presence of comorbidities, and preoperative cardiac function, either in the whole study population or in the subgroup of patients with ACEF scores ≥ 1.1. The type of cardiac surgery and the proportion of urgent operation were similar between the 2 groups. In both the total patient group and the subgroup of patients with ACEF scores ≥ 1.1, those with AKI had a longer bypass time.

**Table 1:** Baseline characteristics of the patients, stratified by AKI after cardiac surgery.

	All patients			ACEF ≥ 1.1		
Characteristics	AKI	Non-AKI	*P*	AKI	Non-AKI	*P*
Patient number	81	96	-	42	31	-
**Preoperative demographic data**						
Age, year	61.1±13.9	59.6±13.5	0.474	66.0±11.6	66.6±11.5	0.834
Male gender, n (%)	51 (63.0)	61 (63.5)	1.000	27 (64.3)	21 (67.7)	0.807
Diabetes mellitus, n (%)	31 (38.3)	30 (31.3)	0.345	22 (52.4)	16 (51.6)	1.000
Hypertension, n (%)	51 (63.0)	50 (52.1)	0.171	24 (57.1)	22 (71.0)	0.327
MI within 6 months, n (%)	17 (21.0)	14 (14.6)	0.322	13 (31.0)	9 (29.0)	1.000
PAD, n (%)	5 (6.2)	3 (3.1)	0.472	4 (9.5)	3 (9.7)	1.000
CHF Fc III/V, n (%)	19 (23.5)	17 (17.7)	0.356	12 (28.6)	10 (32.3)	0.799
Atrial fibrillation, n (%)	17 (21.0)	12 (12.5)	0.155	9 (21.4)	5 (16.1)	0.765
Mechanical ventilation, n (%)	2 (2.5)	0 (0.0)	0.208	2 (4.8)	0 (0.0)	0.505
LVEF, %	58.6±16.0	60.7±15.8	0.368	48.0±13.5	45.3±14.9	0.417
**Preoperative laboratory data**						
Leukocyte count, 1000/ml	7.6±3.6	7.4±2.6	0.688	7.6±3.5	7.6±2.8	0.967
Hemoglobin, g/dl	12.3±2.5	12.9±2.1	0.093	12.0±2.4	12.2±2.3	0.697
Platelet count, 1000/ml	191.0±81.5	205.0±64.8	0.208	195.5±95.9	188.7±69.5	0.740
ALT, u/l	26.7±14.8	32.9±45.9	0.246	24.8±13.4	38.6±71.2	0.223
Creatinine, mg/dl	1.27±1.06	0.88±0.31	0.001	1.51±1.38	1.00±0.38	0.049
Albumin, mg/dl	3.8±0.5	3.9±0.4	0.168	3.7±0.5	3.8±0.5	0.251
**Preoperative scores**						
ACEF score	1.25±0.62	1.09±0.52	0.054	1.67±0.59	1.63±0.59	0.762
STS score (*n* = 144)	6.3±9.8	2.3±3.0	0.001	8.3±11.9	3.7±3.8	0.063
Euro score	12.4±14.1	8.9±11.0	0.068	16.8±17.6	13.6±13.8	0.401
**Surgical detail**						
Urgent operation, n (%)	14 (17.3)	12 (12.5)	0.400	7 (16.7)	4 (12.9)	0.750
Type of heart operation, n (%)			-			-
CABG	25 (30.9)	35 (36.5)		14 (33.3)	14 (45.2)	
Valve surgery	28 (34.6)	38 (39.6)		10 (23.8)	8 (25.8)	
Aorta	16 (19.8)	14 (14.6)		8 (19.0)	5 (16.1)	
Others	1 (1.2)	3 (3.1)		0 (0.0)	0 (0.0)	
CABG + valve surgery	11 (13.6)	6 (6.3)		10 (23.8)	4 (12.9)	
Aortic clamp time (*n* = 117)	130.6±55.7	115.8±38.9	0.097	130.3±58.3	106.8±25.2	0.124
Duration of CPB (*n* = 150)	187.3±91.5	159.1±58.3	0.026	187.2±78.9	137.0±44.6	0.005
**Postoperative biomarker & final outcome**						
NGAL, ng/ml	174.1±203.4	61.3±69.9	<0.001	198.6±187.4	52.4±46.6	<0.001
Renal replacement therapy	12 (14.8)	0 (0.0)	<0.001	8 (19.0)	0 (0.0)	0.018
Extubation hours (*n* = 147)	37.8±47.6	15.1±18.8	<0.001	51.0±58.7	17.1±17.5	0.007
Stay of intensive care unit (days)	5.6±8.5	2.3±1.9	<0.001	6.2±10.8	2.5±1.5	0.067
All-cause mortality, n (%)	9 (11.1)	2 (2.1)	0.024	3 (7.1)	0 (0.0)	0.257

Data are presented as mean ± SD or *n* (%), unless otherwise specified. ACEF: age, creatinine, and ejection fraction; MI: myocardial infarction; PAD: peripheral arterial disease; CHF Fc: congestive heart failure functional class; LVEF: left ventricular ejection fraction; ALT: alanine transaminase; STS: Society of Thoracic Surgeons. CABG: coronary artery bypass grafting; CPB: cardiopulmonary bypass; NGAL: neutrophil gelatinase-associated lipocalin

Compared with the patients without AKI, those with AKI had higher postoperative urinary NGAL levels in both the total patient group (*P* < .001) and the subgroup of patients with ACEF scores ≥ 1.1 (*P* < .001). Eleven patients died within 30 days, with a significantly higher mortality rate in the AKI group than in the non-AKI group (11.1% vs. 2.1%; *P* = .024). Furthermore, the AKI group had longer durations of ventilator use and ICU stay.

### Patient characteristics across ACEF scores

Among the 177 enrolled patients, 73 (41.2%) had ACEF scores ≥ 1.1 and 104 (58.8%) had ACEF scores < 1.1. The baseline characteristics of the 2 groups are presented in Table 2. As expected, compared with patients with ACEF scores < 1.1, those with ACEF scores ≥ 1.1 were significantly older and had higher serum creatinine and lower EF levels. Patients with ACEF scores ≥ 1.1 were more likely to have diabetes mellitus (DM), myocardial infarction within 6 months, PAD, and class III or IV heart failure. Patients with ACEF scores ≥ 1.1 also exhibited higher mean values in Society of Thoracic Surgeons (STS) and Euro scores. No significant differences were observed in the bypass and clamp times.

**Table 2:** Baseline characteristics of the patients, stratified by ACEF scores.

Characteristics	ACEF ≥ 1.1	ACEF < 1.1	*P*
Patient number	73	104	-
**Preoperative demographic data**			
Age, year	66.2±11.5	56.1±13.5	<0.001
Male gender, n (%)	48 (65.8)	64 (61.5)	0.636
Diabetes mellitus, n (%)	38 (52.1)	23 (22.1)	<0.001
Hypertension, n (%)	46 (63.0)	55 (52.9)	0.218
MI within 6 months, n (%)	22 (30.1)	9 (8.7)	<0.001
PAD, n (%)	7 (9.6)	1 (1.0)	0.009
CHF Fc III/V, n (%)	22 (30.1)	14 (13.5)	0.008
Atrial fibrillation, n (%)	14 (19.2)	15 (14.4)	0.416
Mechanical ventilation, n (%)	2 (2.7)	0 (0.0)	0.169
LVEF, %	46.9±14.1	68.8±9.6	<0.001
**Preoperative laboratory data**			
Leukocyte count, 1000/ml	7.6±3.2	7.4±3.0	0.725
Hemoglobin, g/dl	12.1±2.4	13.0±2.3	0.012
Platelet count, 1000/ml	192.6±85.2	202.8±63.2	0.363
ALT, u/l	30.7±47.5	29.6±23.4	0.840
Creatinine, mg/dl	1.29±1.10	0.89±0.34	0.001
Albumin, mg/dl	3.8±0.5	3.9±0.5	0.131
**Preoperative scores**			
ACEF score	1.65±0.59	0.82±0.17	<0.001
STS score (*n* = 144)	6.3±9.6	2.4±4.2	0.001
Euro score	15.4±16.1	7.1±7.9	<0.001
**Surgical detail**			
Urgent operation, n (%)	11 (15.1)	15 (14.4)	1.000
Type of heart operation, n (%)			-
CABG	28 (38.4)	32 (30.8)	
Valve surgery	18 (24.7)	48 (46.2)	
CABG + valve surgery	14 (19.2)	3 (2.9)	
Aorta	13 (17.8)	17 (16.3)	
Others	0 (0.0)	4 (3.8)	
Aortic clamp time (*n* = 117)	121.4±49.5	123.7±47.4	0.804
Duration of CPB (*n* = 150)	167.3±71.4	177.4±82.2	0.433
**Postoperative biomarker and final outcome**			
NGAL, ng/ml	135.7±161.3	96.6±151.9	0.104
Renal replacement therapy	8 (11.0)	4 (3.8)	0.075
Extubation hours (*n* = 147)	37.1±49.2	17.2±20.9	0.001
Stay of intensive care unit (days)	4.6±8.4	3.2±3.7	0.139
All-cause mortality, n (%)	3 (4.1)	8 (7.7)	0.529
AKI, n (%)	42 (57.5)	39 (37.5)	0.010

Data are presented as mean ± SD or *n* (%), unless otherwise specified. ACEF: age, creatinine, and ejection fraction; MI: myocardial infarction; PAD: peripheral arterial disease; CHF Fc: congestive heart failure functional class; LVEF: left ventricular ejection fraction; ALT: alanine transaminase; STS: Society of Thoracic Surgeons. CABG: coronary artery bypass grafting; CPB: cardiopulmonary bypass; NGAL: neutrophil gelatinase-associated lipocalin.

Postoperative urinary NGAL values did not differ between the 2 groups (*P* = .104). The incidence of AKI was significantly higher among the patients with ACEF scores ≥ 1.1 than among those with ACEF scores < 1.1 (57.5% vs. 37.5%, *P* = .010). A trend of a higher incidence of dialysis-requiring AKI was observed in the patients with ACEF scores ≥ 1.1 (11.0% vs. 3.8%, *P* = .075).

### Performance of NGAL in discriminating AKI

**Fig 1:**
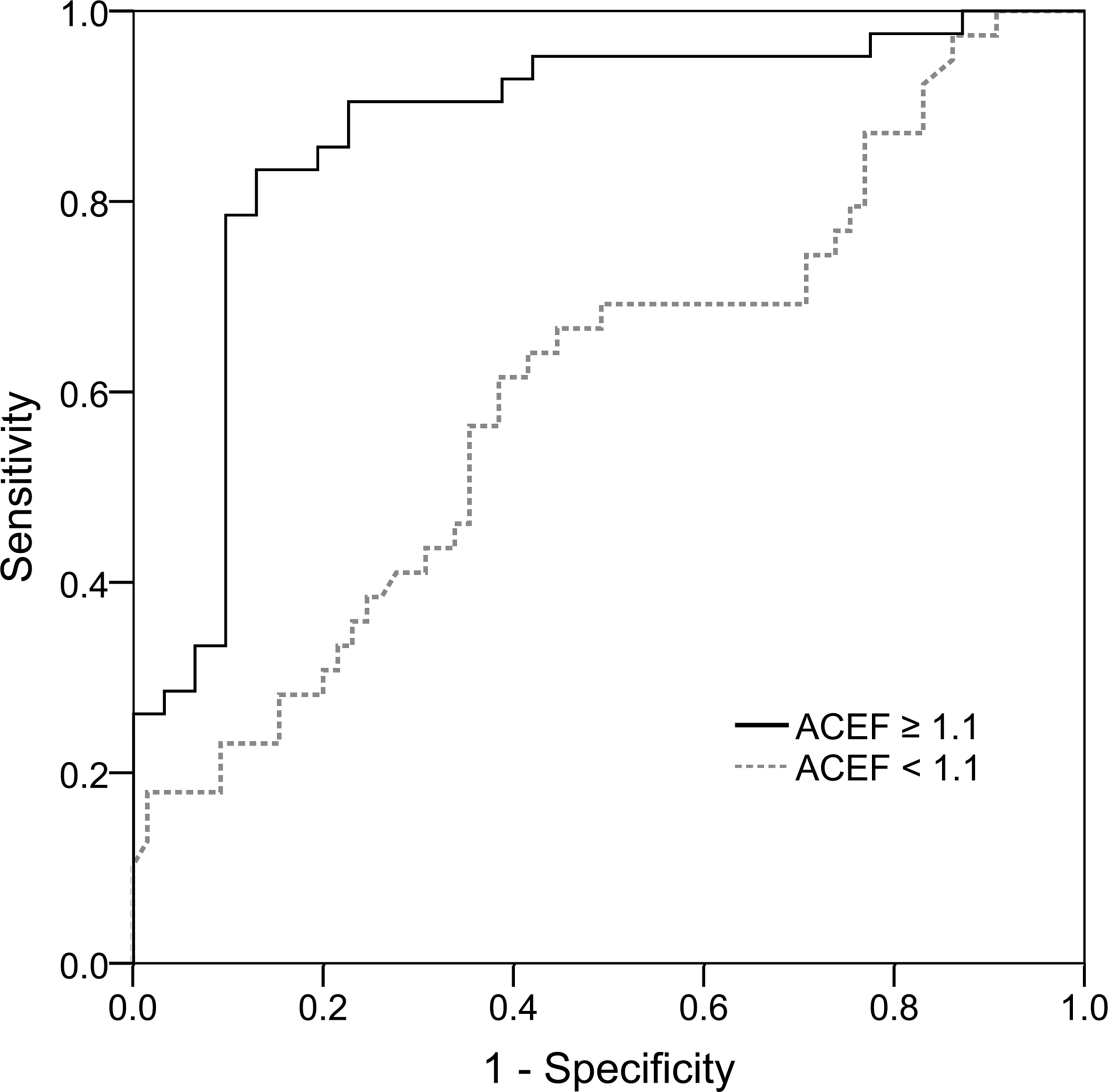
AUROC for NGAL levels in discriminating AKI stratified by ACEF score. AUROCs were 0.606 (0.492–0.720) for ACEF scores < 1.1 and 0.873 (0.784–0.961) for ACEF scores ≥ 1.1, with a significant difference in the values (*P* < .001).

We then examined the performance of the urinary NGAL test for diagnosing AKI. As shown in Table 3, urinary NGAL exhibited satisfactory discrimination ability in the whole study population, with an AUROC of 0.732 (95% confidence interval [CI], 0.656–0.808). The discrimination ability of urinary NGAL for predicting AKI in individuals with ACEF scores ≥ 1.1 (AUROC, 0.873; 95% CI, 0.784–0.961) outperformed that in individuals with ACEF scores < 1.1 (AUROC, 0.606; 95% CI, 0.492–0.720), with a significant difference in the AUROC (*P* < .001; Fig 1). The optimal cutoff value of urinary NGAL in the total cohort was 82.6 ng/mL, with a sensitivity of 60.5% and a specificity of 80.2%. By contrast, urinary NGAL exhibited a higher sensitivity of 83.3% and specificity of 87.2% for a cutoff value of 77.5 ng/mL when applied in patients with ACEF scores ≥ 1.1. Noticeably, when the population incidence of AKI was set at 40% [4,5,20], the obtained positive predictive value was 81.2% and negative predictive value was 88.7%, given the cutoff value of 77.5 ng/mL in patients with ACEF scores ≥ 1.1.

**Table 3:** Performance of NGAL in discriminating AKI, stratified by ACEF scores.

Population	AUROC (95% CI)	*P* value	Optimal cut-off†	Sensitivity (%)	Specificity (%)	PPV§ (%)	NPV§ (%)
ACEF < 1.1	0.606 (0.492–0.720)	0.071	>45.1	61.5	61.5	50.5	68.5
ACEF ≥ 1.1	0.873 (0.784–0.961)	<0.001	>77.5	83.3	87.2	81.2	88.7
Total	0.732 (0.656–0.808)	<0.001	>82.6	60.5	80.2	67.1	75.3

† According to the Youden index

§ Incidence of AKI was estimated to be approximately 40%, with 28.9% for CABG (reference 5), 38.7% for valve (reference 4), and 52.7% for aorta (reference 20)

NGAL: neutrophil gelatinase-associated lipocalin; AKI: acute kidney injury; ACEF: age, creatinine, and ejection fraction; AUROC: area under the receiver operating characteristic curve; CI: confidence interval; PPV: positive predictive value; NPV: negative predictive value.

**Fig 2:**
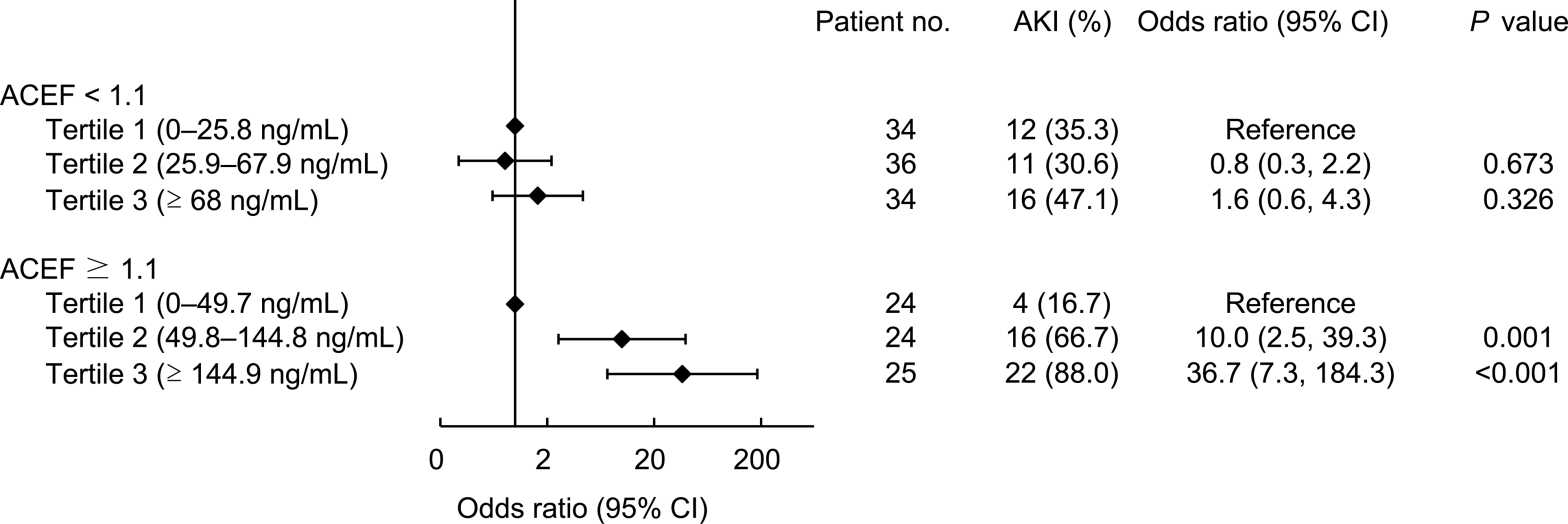
Incidence of AKI across the tertiles of NGAL levels, stratified by ACEF score.

Fig 2 depicts the incidence of AKI across the tertiles of urinary NGAL, stratified by the ACEF score. Notably, the incidence of AKI in patients with ACEF scores ≥ 1.1 stratified by urinary NGAL tertiles increased from 16.7% to 66.7% to 88.0% in the first, second, and third tertiles, respectively, and a significant increasing trend was observed. The odds ratios for the development of AKI in patients with ACEF scores ≥ 1.1 were 10.0 and 36.7 for the second and third tertiles of urinary NGAL compared with those for the lowest tertile. By contrast, the risk for the occurrence of AKI was not increased across urinary NGAL tertiles in patients with ACEF scores < 1.1.

## Discussion

In this study, we demonstrated the performance characteristics of a two-step diagnostic framework, combined risk assessment through ACEF scores first and then urinary NGAL test, a urinary biomarker of tubular injury measured within 6 hours after cardiac surgery, for detecting subsequent AKI. We discovered that simple preoperative risk stratification by ACEF scores ≥ 1.1, followed by urinary NGAL levels, offered significant complementary risk stratification with superior discrimination.

AKI remains a major complication in patients undergoing cardiac surgery. AKI incurs significant costs and is independently associated with adverse clinical outcomes.[5, 6, 21] Accumulating evidence has revealed that the distinctive characteristics of cardiac surgery, including cardiopulmonary bypass and aortic clamping, which compromise renal perfusion and induce systemic inflammatory response, contribute substantially to the development of AKI.[8, 22–24] Our data are consistent with these findings. Our study demonstrated that the patients in the AKI group had a higher mortality rate than did those in the non-AKI group (11.1% vs. 2.1%; *P* = .024). The patients in the AKI group had a longer bypass time than did those in the non-AKI group (187.3 ± 91.5 minutes vs. 159.1 ± 58.3 minutes; *P* = .026). Because AKI is a well-established risk factor for mortality in patients undergoing cardiac surgery, earlier identification of patients at risk for AKI would allow physicians to initiate interventions to impede disease progression. The usual 24–36-hour delay in serum creatinine rise after a definite renal insult implies that serum creatinine is not a sufficiently sensitive parameter for the measurement of kidney function in AKI.[12, 25, 26]

Our study demonstrated that increases in urinary NGAL levels after cardiac surgery anticipated the subsequent development of AKI (as defined by the KDIGO criteria), with an AUROC of 0.732 (95% CI, 0.656–0.808). This finding is consistent with previous results from the Translational Research Investigating Biomarker End-Points in Acute Kidney Injury (TRIBE-AKI) study[27] and other investigations,[11, 12, 28–30] which have demonstrated that postoperative urinary NGAL is an effective biomarker for early identification of AKI. The TRIBE-AKI study tested the ability of several biomarkers, including urinary NGAL, to detect AKI in 1219 adults who underwent cardiac surgery. The AUROCs of urinary NGAL in this cohort at 0–6 and 6–12 hours were 0.67 and 0.70, respectively.[27] Similarly, in a prospective study of 85 adult patients who underwent cardiac surgery, Matsui et al. stated that the AUROC was 0.74 for predicting the development of AKI according to urinary NGAL levels at 6 hours postoperatively.[29] NGAL is a 25-kDa protein originally identified from human neutrophils as a shuttle for iron transport,[31] and its upregulation has been demonstrated in the proximal tubule shortly after exposure to harmful insults such as ischemia or nephrotoxins.[32, 33] The resultant increased NGAL synthesis is released by injured tubular epithelial cells within hours after AKI. Crucially, both the expression of NGAL in the injured renal tubule and the concentration of NGAL in the urine increase in proportion to the severity of kidney injury,[12, 34, 35] thus providing biological plausibility for its use as an AKI biomarker.

Current evidence does not support routine clinical use because of its moderate discrimination ability and variable thresholds (cutoffs).[14] In addition, unrestrictedly screening every case is not financially feasible. Therefore, risk stratification by robust clinically validated models, followed by biomarkers, should be considered a more optimal and cost-efficient strategy in daily care. Our previous studies have revealed that ACEF scores are a suitable tool for predicting AKI in patients undergoing either CABG[5] or mitral valve surgery[4], with discrimination ability comparable to that of other scoring systems such as STS and Euro scores. Both STS and Euro scores consist of complicated mathematical calculations and require a web-based application to enhance implementation. By contrast, ACEF scores are easy to apply, with a balance between predictability and simplicity. In the current study, patients with ACEF scores ≥ 1.1 exhibited higher mean STS and Euro scores and demonstrated a higher incidence of AKI. These findings contribute to the evidence on the prognostic utility of ACEF scores, and we report another critical finding: risk stratification by ACEF scores raised the AUROC of urinary NGAL from 0.732 to 0.873 for predicting AKI. When the cutoff value of urinary NGAL was set at 77.5 ng/mL, the sensitivity was 83.3%, specificity was 87.2%, and negative predictive value was 88.7%. This superior discrimination ability of urinary NGAL after risk stratification by ACEF scores has 2 possible explanations. First, clinical assessment through ACEF scores enriched the population for the development of AKI and thus largely improved the sensitivity (from 60.5% to 83.3%) and overall performance of urinary NGAL. Indeed, our study revealed that patients with ACEF scores ≥ 1.1 were older and more likely to have DM, myocardial infarction, PAD, and severe heart failure, which are well-established risk factors for AKI.[7, 8, 23] Another explanation is that patients with ACEF scores < 1.1 have probably received damage to their kidneys (as demonstrated by the similar levels of urinary NGAL to that in patients with ACEF scores ≥ 1.1), but not of sufficient severity to cause any subsequent rise in serum creatinine. The exclusion of these populations helps to improve or retain the specificity (from 80.2% to 87.2%) of urinary NGAL for predicting AKI.

Some studies have identified baseline renal function as a possible modifier of the discrimination ability of urinary NGAL as an AKI biomarker, with greater predictive ability in patients with eGFR ≥ 60 mL/min than in those with eGFR < 60 mL/min.[36, 37] This may be due to the less-functional nephron mass with a reduced ability to produce NGAL during renal insult. However, our study demonstrated that patients with ACEF scores ≥ 1.1 had higher serum creatinine values, namely lower eGFRs, than did those with ACEF scores < 1.1. Thus, baseline renal function was unlikely to have contributed appreciably to our favorable discriminatory power of urinary NGAL among patients with ACEF scores ≥ 1.1 in the present series.

We are not aware of previous studies investigating the performance of AKI biomarkers with a two-step diagnostic framework combining risk stratification by a clinically validated model as the first line. However, a few reports have incorporated multiple variables, including one biomarker, for predicting AKI after cardiac surgery. The TRIBE-AKI study combined a biomarker and clinical model, and found that adding clinical model significantly but only modestly raised the AUROC of urine IL-18 and plasma NGAL from 0.74 to 0.76 (*P* = .03) and 0.70 to 0.75 (*P* = .01), respectively. Combining urinary NGAL and their clinical model failed to significantly improve model discrimination (*P* = .12).[27] The difference may be attributable to a different diagnostic framework for the study. Another possible explanation is that different clinical models were used. Our study used a simple validated risk score comprising only 3 preoperative variables, namely age, preoperative creatinine, and ejection fraction. However, the clinical model adopted in the TRIBE-AKI study comprised 8 self-constructed variables, including 7 preoperative variables (age, preoperative eGFR, race, sex, DM, hypertension, and urgent surgery) and one intraoperative variable (bypass time > 120 minutes). Certain factors, such as urgent surgery and bypass time > 120 minutes, could be associated with both increased postoperative urinary NGAL levels and increased risk of AKI, leaving little room for additional improvement. Additional studies are warranted to investigate this possibility.

Although many studies investigating urinary NGAL utility in predicting the development of AKI have normalized NGAL against creatinine to control changes in urine flow rate, the underlying assumption of constant creatinine excretion may be flawed.[38, 39] Lower creatinine excretion in the setting of acute GFR loss during early stage of AKI may amplify the NGAL performance irrespective of whether NGAL synthesis is increased by injury or not. However, we analyzed our data using normalized values and the results are consistent with those using absolute NGAL values. Risk stratification by ACEF scores ≥ 1.1, followed by normalized NGAL, still provides more satisfactory risk discrimination (AUROC, 0.876; 95% CI, 0.800–0.953) than does normalized NGAL alone (AUROC, 0.754; 95% CI, 0.681–0.826) for the early detection of AKI.

This study has several limitations. First, despite how the time course of change depicted that NGAL peaked at approximately 4 hours after cardiopulmonary bypass,[12, 36] repeated measurements might further improve the discrimination. Second, in this study, AKI was identified within 7 days, and postoperative care, postoperative bleeding, and infection also contributed to the AKI; therefore, caution is warranted when expanding the application of our findings to AKI occurring at 7 days after surgery. Third, we did not include urine output criteria in the AKI definition, which might lead to underestimate AKI incidence. Four, the etiologies of AKI might vary in different types of cardiac surgery. The relatively small sample size of this study prevented an adequate subgroup analysis, and thus, the applicability of our data to a specific type of cardiac surgery is limited.

In conclusion, the results of the current study indicated that ACEF scores can help to identify patients with a higher risk of AKI after cardiac surgery. A two-step diagnostic framework combining preoperative risk assessment through ACEF scores first, followed by postoperative urinary NGAL evaluation, provide more accurate risk discrimination than does urinary NGAL alone for the early detection of AKI after cardiac surgery. Additional studies are required to investigate whether this strategy could improve outcomes and reduce the costs of care in patients undergoing cardiac surgery.
